# Supplementary figures and images for: Expression and role of lumican in acute aortic dissection: A human and mouse study
Source: PLoS One. 2021 Jul 26;16(7):e0255238. doi: 10.1371/journal.pone.0255238 (PMC8312931; doi:10.1371/journal.pone.0255238)

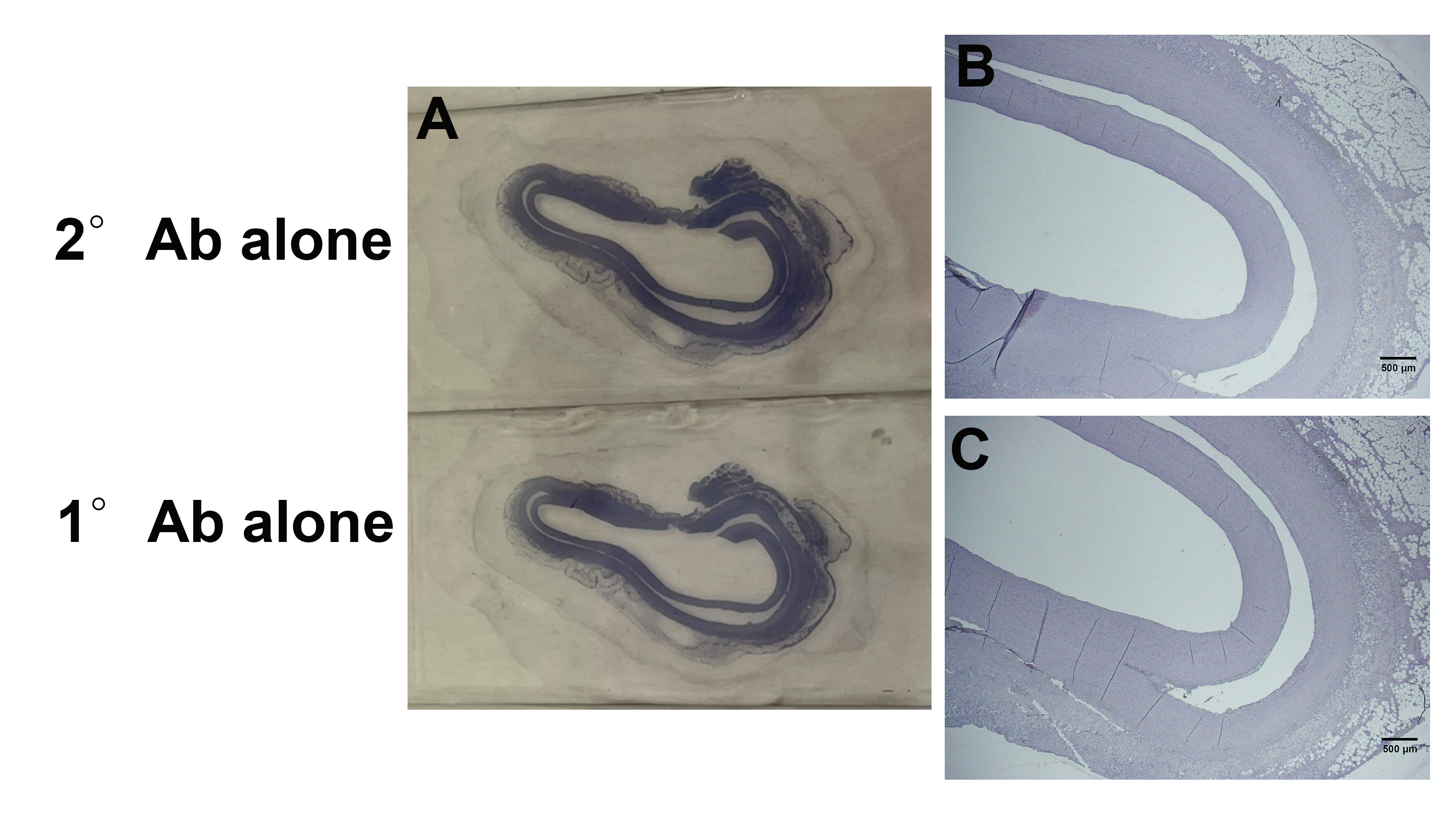

Supplement: S1 Fig — (TIF) [file pone.0255238.s001.tif]

Fig. 6A

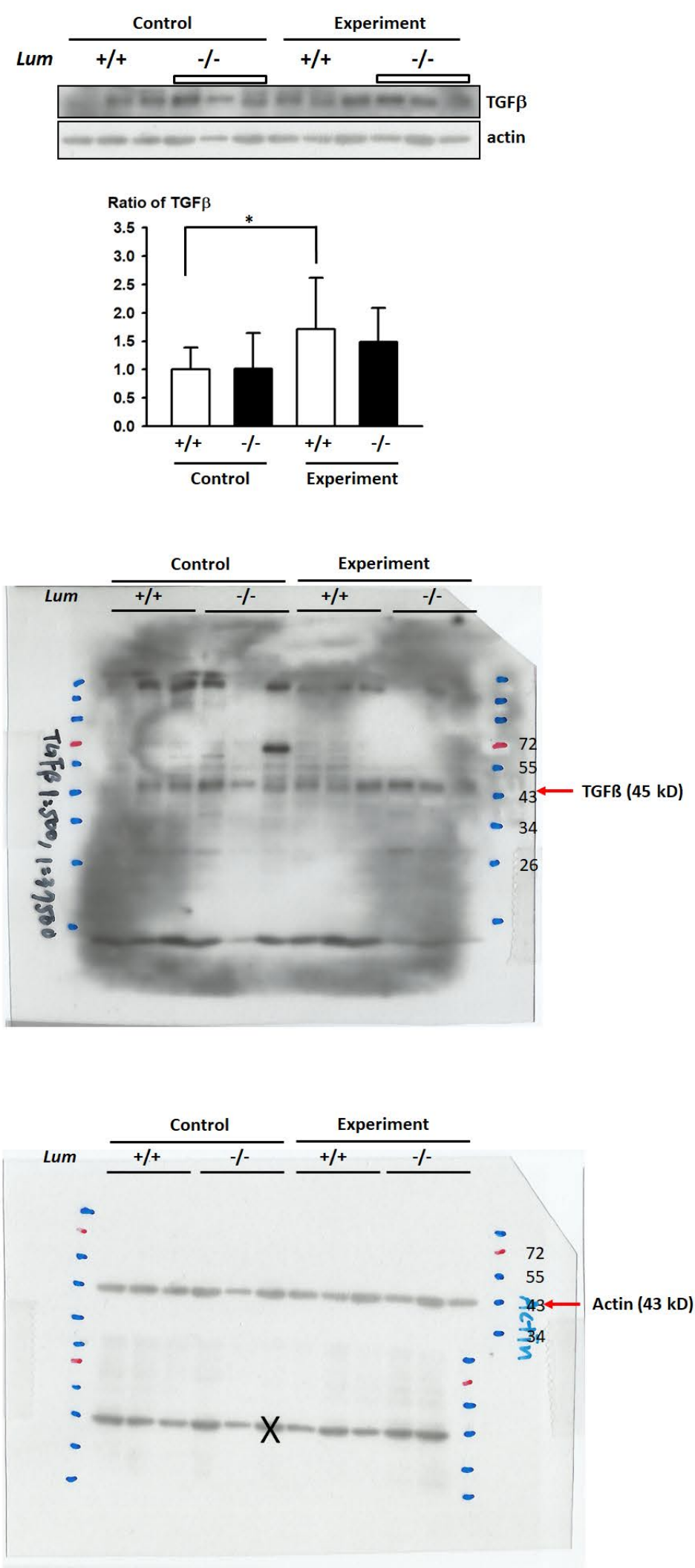

Fig. 6B

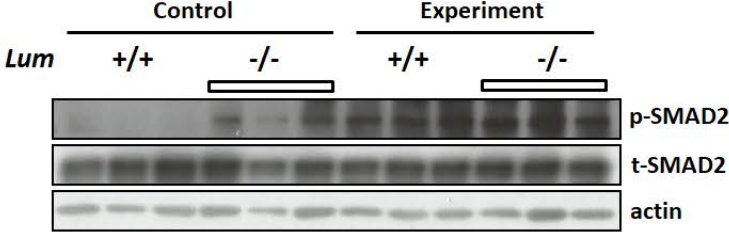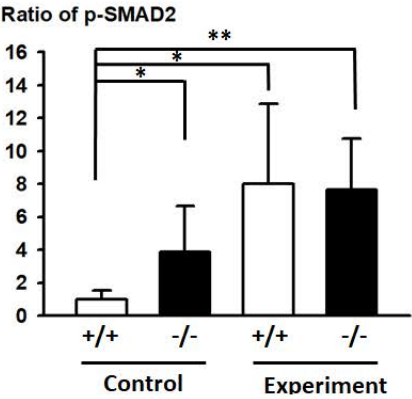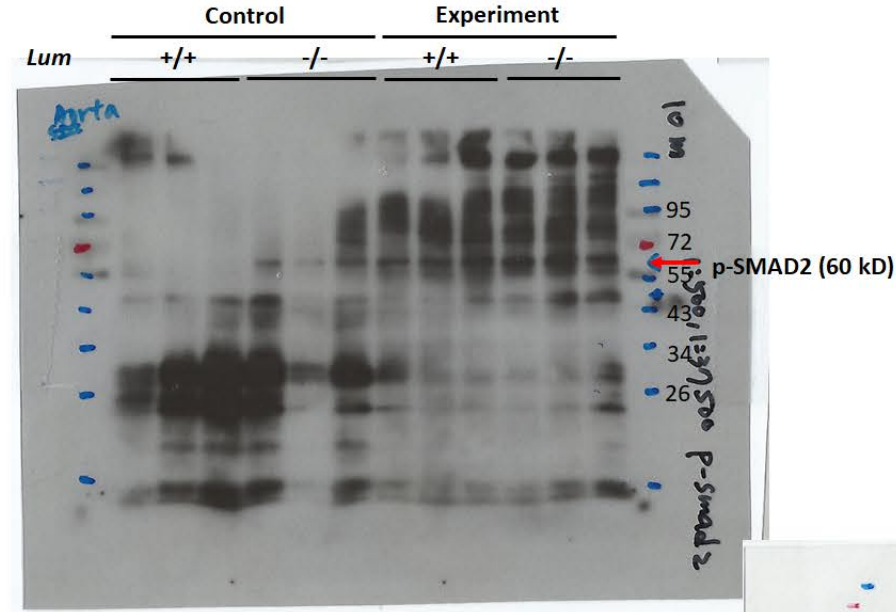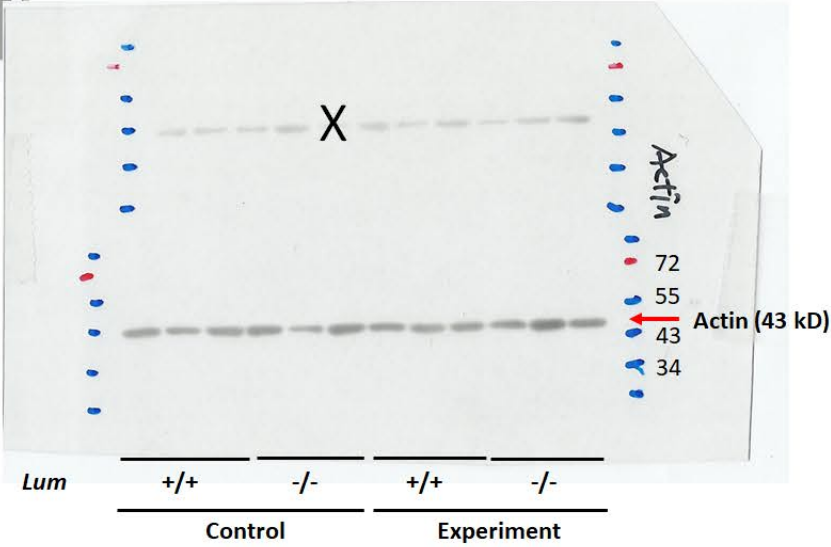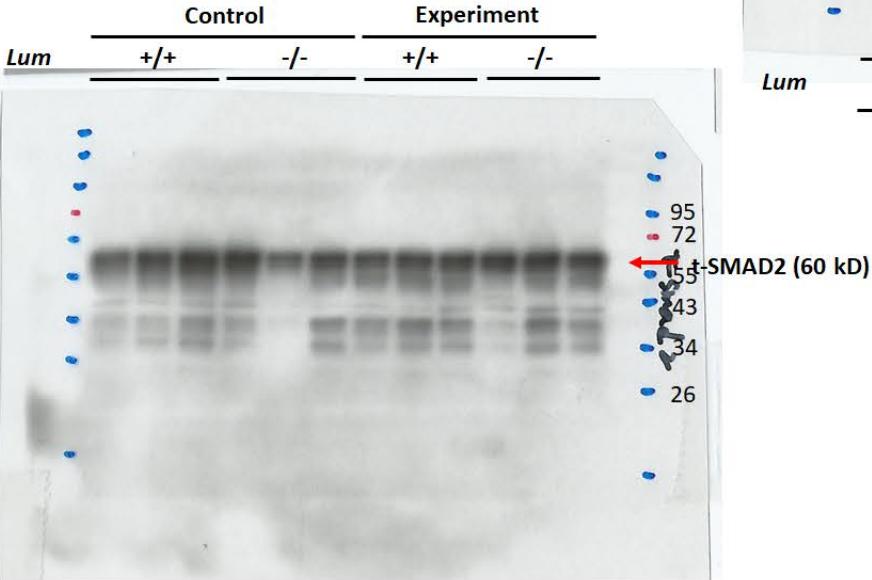

Fig. 6C

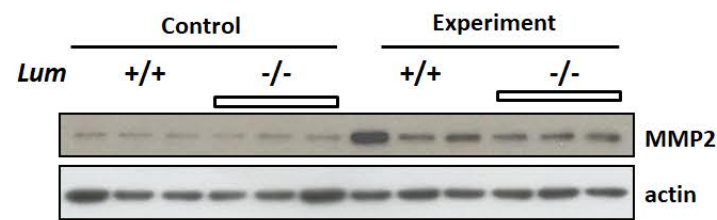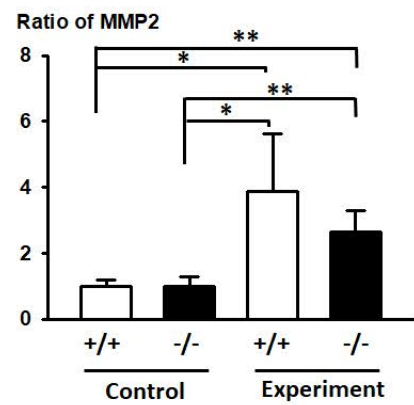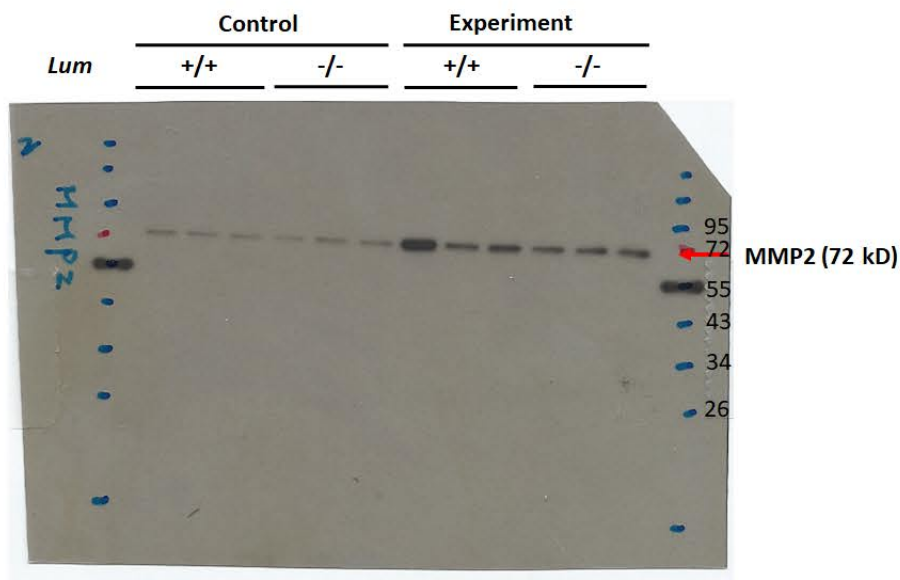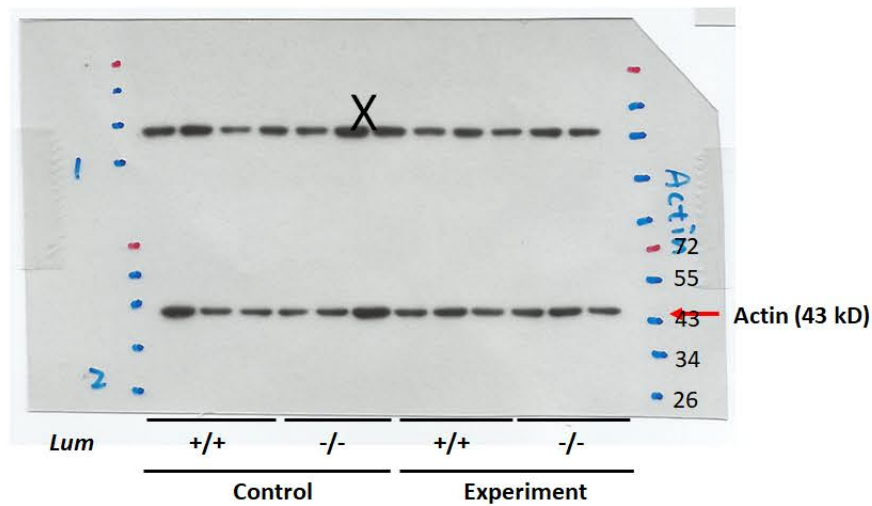

Fig. 6D

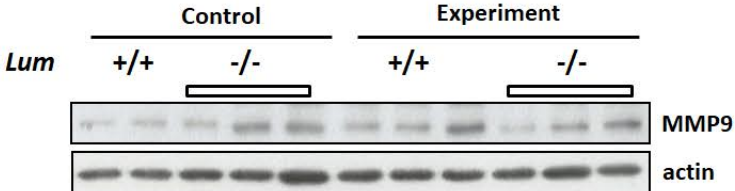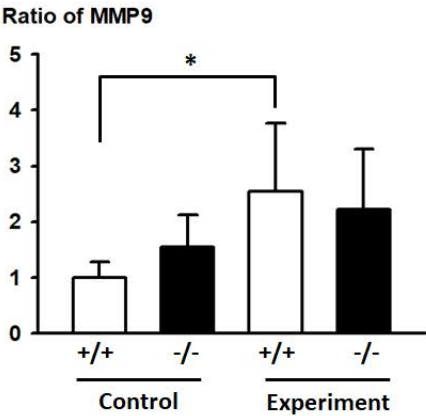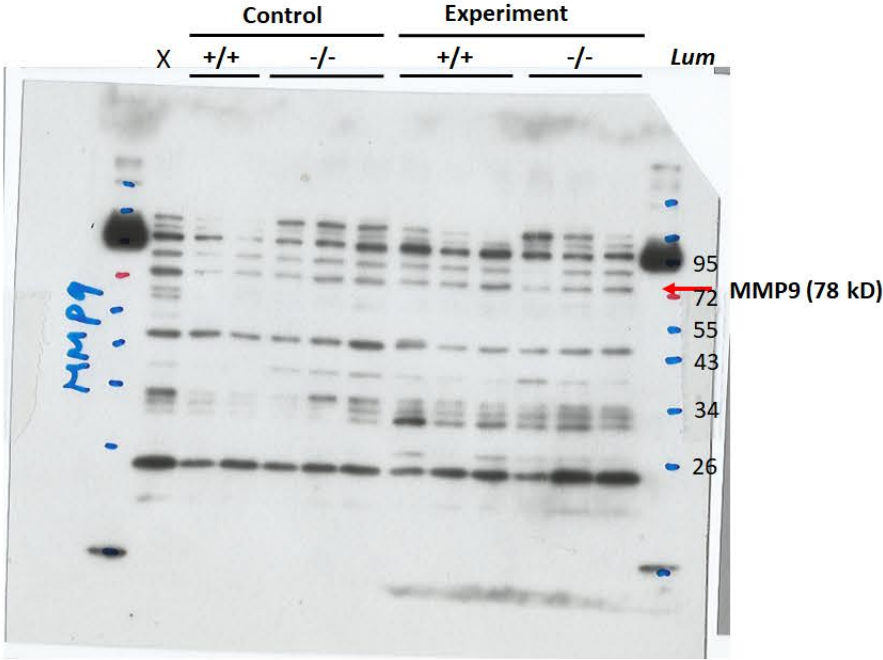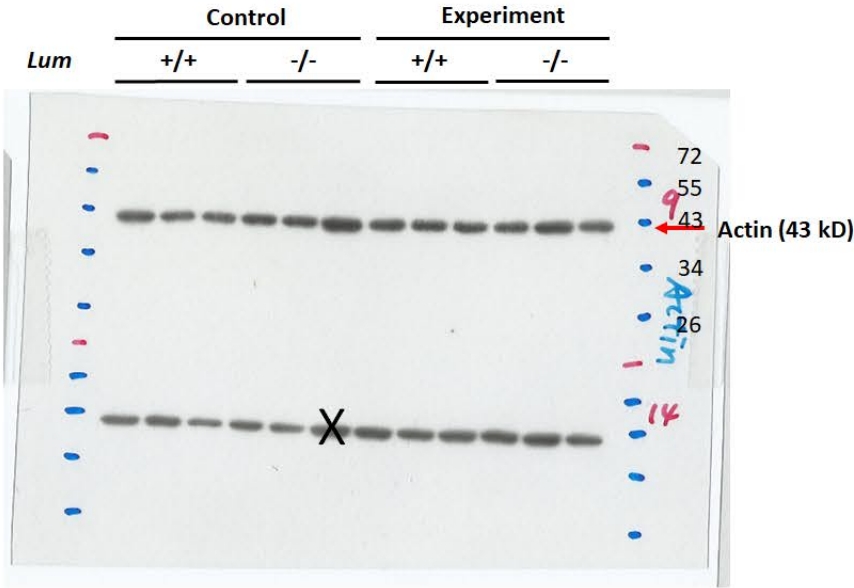

Fig. 6E

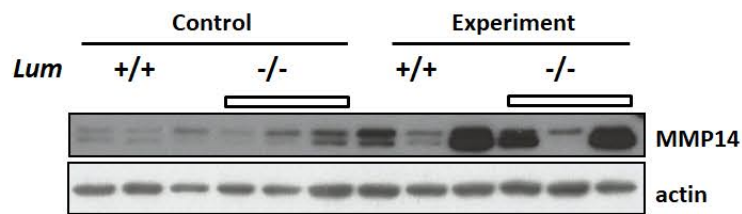

Ratio of MMP14

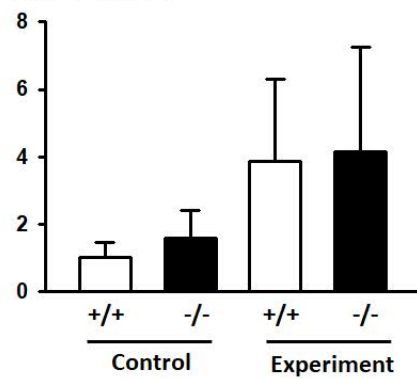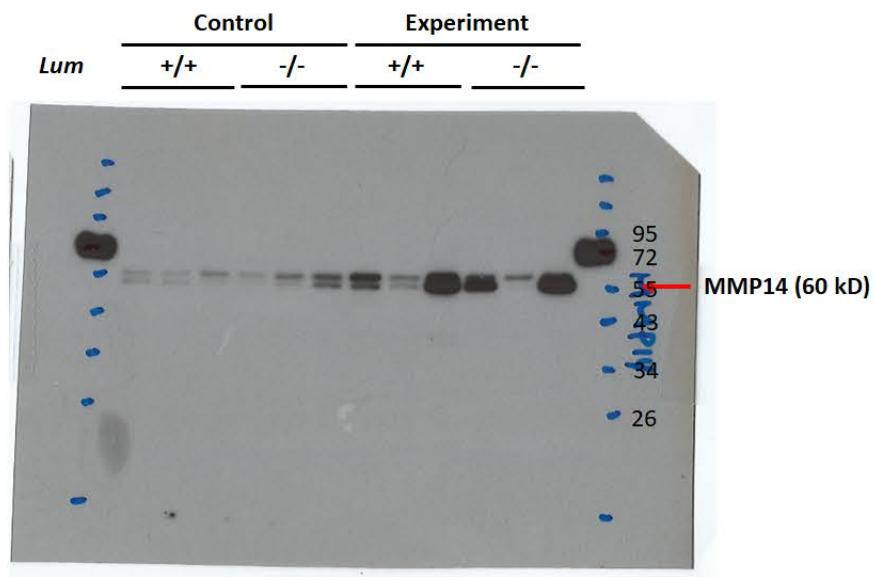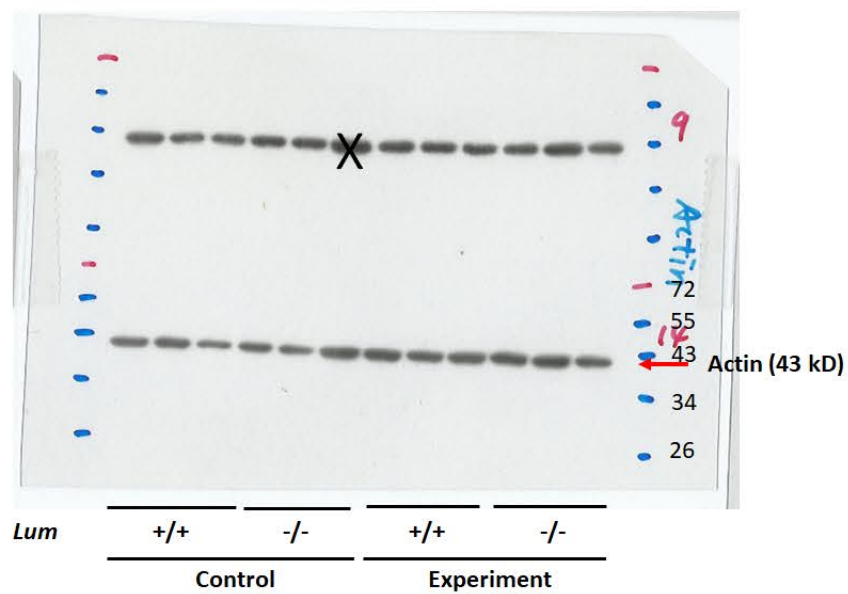

Fig. 6F

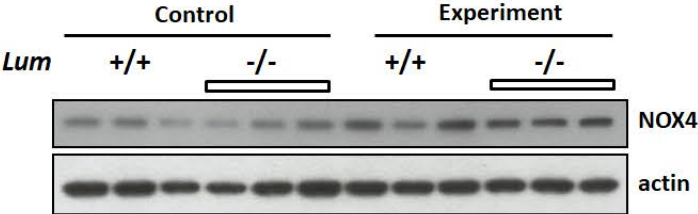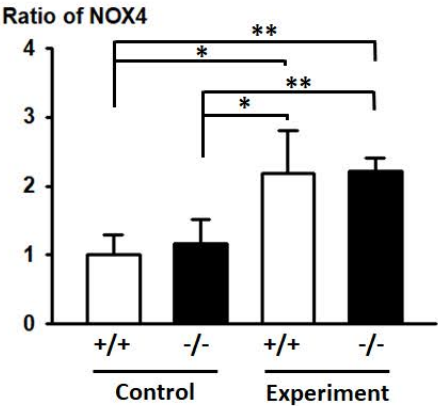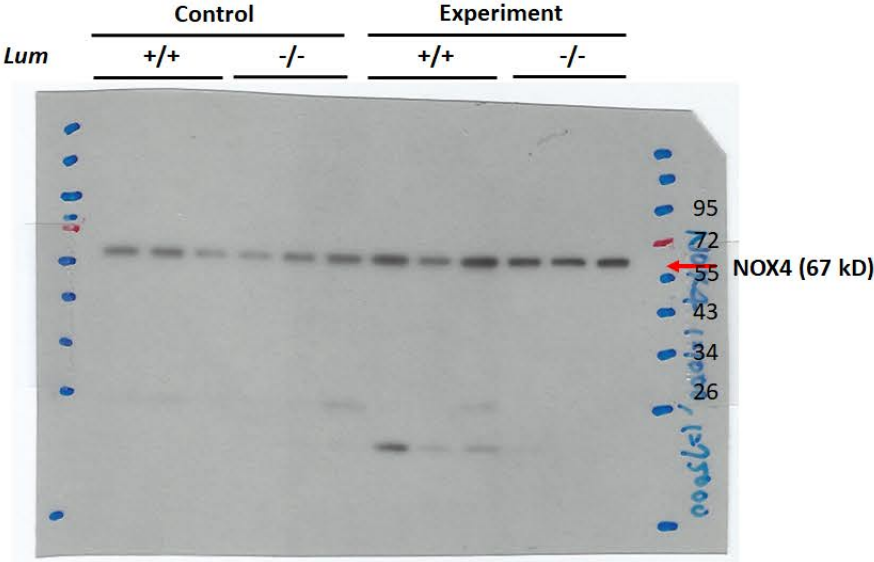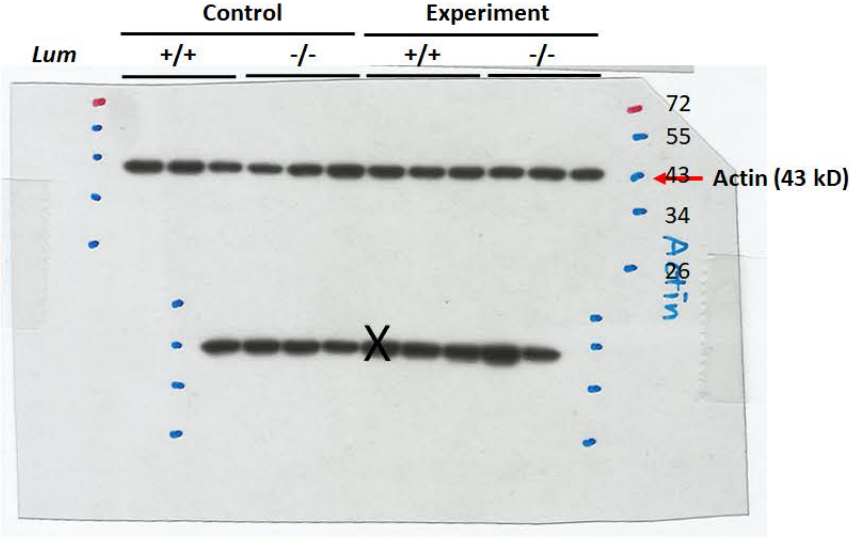

Fig. 7

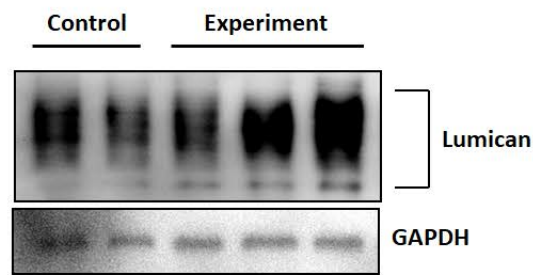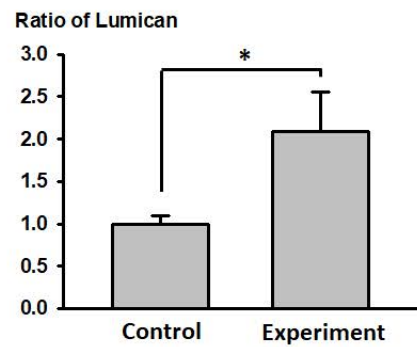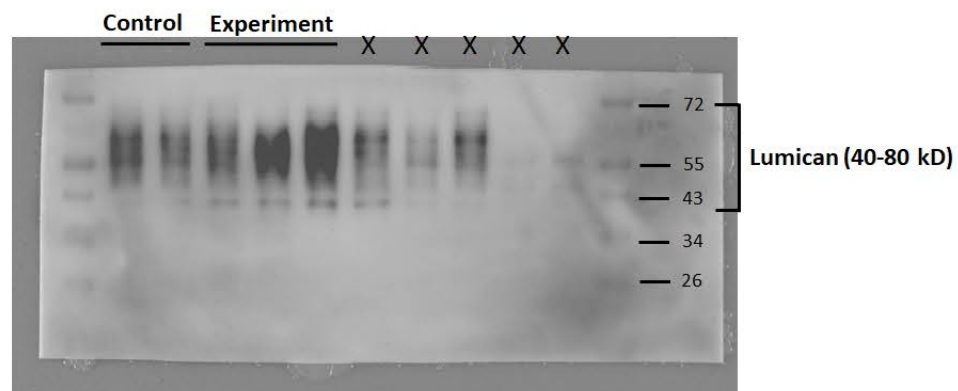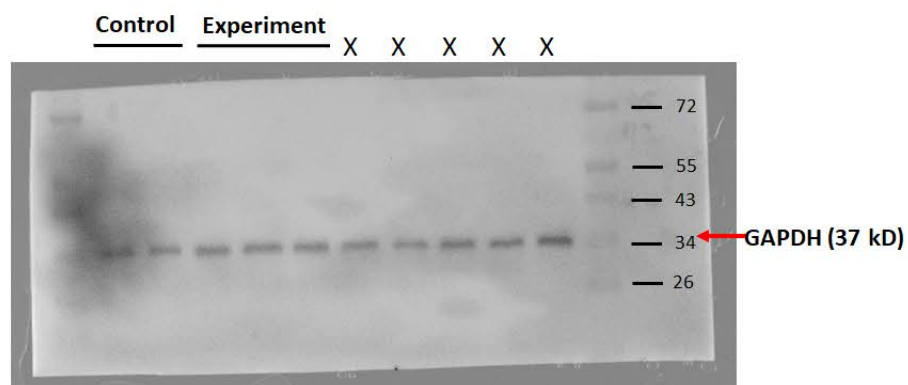

Supplement: S1 Raw images — (PDF) [file pone.0255238.s002.pdf]
