# Supplementary material for: Expression and role of lumican in acute aortic dissection: A human and mouse study
Source: PLoS One. 2021 Jul 26;16(7):e0255238. doi: 10.1371/journal.pone.0255238 (PMC8312931; doi:10.1371/journal.pone.0255238)
Supplement: Attachment — Submitted filename: Chen et al_PLosOne_2021.docx [file pone.0255238.s003.docx]

The manuscript entitled "Expression and Role of Lumican in Acute

Aortic Dissection: A Human and Mouse Study" by Chen et al examined LUM expression 40 patterns in patients with AD and explored the molecular functions of LUM in a mouse AD model. The authors concluded that LUM expression is altered in patients with AAD, and Lum−/− mice exhibited augmented AD pathogenesis and LUM may be a novel target for AD therapy.

While the concept of the paper is of high importance towards the possibility of Lum as novel therapeutic target for AAA, There are major concerns with the manuscript:

1. While differences in Lum expression was observed between acute AD (AAD) and chronic AD (CAD), it is not clear how these tissue samples was characterized. Also, in Fig. 1, the staining is not clear and resolution is poor. Infact, in Fig. 1F, it seems that expression of Lum is increased in the adventitial layer.

2. Figs. 4C-F in the present form is not adding much to the data. The authors need to add high magnification images and interpretation of the results.

3. The authors reported higher TAD in the Lum KO mice, how does it correlate to the human AADs is not clear.

4. It seems that most of the mice (18/22) died of TAD. It is not clear how the remaining mice were used.

5. It is not clear which part of the tissue (aorta?) and how many were used for Fig 6A. It is also not clear whether the authors used abdominal or the whole aorta.
