# Supplementary material for: Expression and role of lumican in acute aortic dissection: A human and mouse study
Source: PLoS One. 2021 Jul 26;16(7):e0255238. doi: 10.1371/journal.pone.0255238 (PMC8312931; doi:10.1371/journal.pone.0255238)
Supplement: Attachment — Submitted filename: Response to Reviewers.docx [file pone.0255238.s005.docx]

July 1, 2021

Editor-in-Chief
*PLOS ONE*

PONE-D-21-03961 Expression and Role of Lumican in Acute Aortic Dissection: A Human and Mouse Study

Dear Editor:

On behalf of the authors of this manuscript, we thank you, associate editor and 2 reviewers very much for your thorough review of this manuscript. Your suggestions were very thoughtful and instructive. The reviewers identified some serious and important concerns that we have not considered while making this study. We revised the manuscript according to the comments of the reviewers’ point-by-point as follows. We hope that the revised manuscript will retain your attention and you will judge the revised manuscript to be suitable for publication in “*PLOS ONE*”. Please forward the revised manuscript to the reviewers and reconsider the work for publication.

Yours sincerely,

First authors: Shao-Wei Chen

Corresponding author: Pao-Hsien Chu

Reviewer #1: The authors have been responsive to the critiques. The revised manuscript is much improved. Here are minor comments:
Fig 1 and Fig 4. In figure legends, please indicate the size of the bar. The size in the figures are too small to read.

**Reply: We have revised the size bar in Fig 1 and Fig 4, and we also indicated the size of the bar in the figure legend.**

It is interesting that the expression of Lum is increased in the aorta. Since AD occurs preferentially at the ascending aorta, do you see differential expression of Lum in thoracic and abdominal aorta?

**Reply: Thank you for your valuable comment. Unfortunately,** **because of the limited number of WT mice with AD in the experimental group, we could not obtain enough samples to explore the different expressions of Lum in the thoracic and abdominal aorta.** **We will further explore this interesting issue in our future study. Thanks again.**

Reviewer #2: The authors have addressed all the concerns satisfactorily. The Reviewer does not have additional concerns.

**Reply: Thank you for your review.**
